# Acute effects of empagliflozin on open-loop baroreflex function and urine glucose excretion in Goto-Kakizaki diabetic rats

**DOI:** 10.1186/s12576-023-00861-9

**Published:** 2023-04-12

**Authors:** Toru Kawada, Hiromi Yamamoto, Aimi Yokoi, Akitsugu Nishiura, Midori Kakuuchi, Shohei Yokota, Hiroki Matsushita, Joe Alexander, Keita Saku

**Affiliations:** 1 Department of Cardiovascular Dynamics, National Cerebral and Cardiovascular Center, Osaka, 564-8565 Japan; 2 Department of Cardiovascular Medicine, Kurashiki Central Hospital, Ohara HealthCare Foundation, Okayama, 710-8602 Japan; 3 Medical and Health Informatics, NTT Research, Inc., Sunnyvale, CA 94085 USA

**Keywords:** Sodium–glucose cotransporter 2, Sympathetic nerve activity, Arterial pressure, Urine flow, Equilibrium diagram

## Abstract

Although suppression of sympathetic activity is suggested as one of the underlying mechanisms for the cardioprotective effects afforded by sodium–glucose cotransporter 2 (SGLT2) inhibitors, whether the modulation of glucose handling acutely affects sympathetic regulation of arterial pressure remains to be elucidated. In Goto–Kakizaki diabetic rats, we estimated the open-loop static characteristics of the carotid sinus baroreflex together with urine glucose excretion using repeated 11-min step input sequences. After the completion of the 2nd sequence, an SGLT2 inhibitor empagliflozin (10 mg kg^−1^) or vehicle solution was administered intravenously (n = 7 rats each). Empagliflozin did not significantly affect the baroreflex neural or peripheral arc, despite significantly increasing urine glucose excretion (from 0.365 ± 0.216 to 8.514 ± 0.864 mg·min^−1^·kg^−1^, P < 0.001) in the 7th and 8th sequences. The possible sympathoinhibitory effect of empagliflozin may be an indirect effect associated with chronic improvements in renal energy status and general disease conditions.

## Background

Empagliflozin increases urine glucose excretion by inhibiting sodium–glucose cotransporter 2 (SGLT2) at renal proximal tubules. Although empagliflozin was developed to treat diabetic mellitus (DM), the EMPA-REG OUTCOME trial revealed that empagliflozin can exert a beneficial effect on cardiovascular diseases [1]. The cardiovascular effects are considered to be a class effect of SGLT2 inhibitors [2] and independent of the presence of DM [3, 4]. Several mechanisms are postulated for the beneficial cardiovascular effects of SGLT2 inhibitors such as the attenuation of glucotoxicity, improvements in the loading conditions of the heart, and improvements in cardiac metabolism via increased ketone bodies [5, 6]. Sympathetic suppression is also suggested because reflex tachycardia, which occurs in response to a volume reduction by diuretics, does not occur during treatment with SGLT2 inhibitors [7–9].

Glucose reabsorption via SGLT2 depends on the sodium gradient across the plasma membrane, which is maintained by Na^+^/K^+^ ATPase activity. The ATP level in the proximal tubules decreases within 2 min after renal ischemia [10], suggesting a high metabolic rate of the kidneys. In DM, renal proximal tubular cells are overloaded by excessive glucose reabsorption, and SGLT2 inhibitors can reduce the energy consumption. There is a possible link between renal energy expenditure and systemic sympathetic activation [2]. For instance, oxygen deficiency or enhanced organ work promotes the generation of adenosine following ATP breakdown [11], and adenosine can trigger renal afferent signaling for sympathoexcitatory reflexes [12]. It remains unknown, however, whether the modulation of glucose handling by SGLT2 inhibitors acutely affects baroreflex-mediated sympathetic arterial pressure (AP) regulation. The present study examined the acute effects of empagliflozin on baroreflex open-loop static characteristics and urine glucose excretion in anesthetized Goto–Kakizaki (GK) diabetic rats.

## Methods

### Ethical approval

Animals were cared for in strict accordance with the Guiding Principles for the Care and Use of Animals in the Field of Physiological Sciences, which has been approved by the Physiological Society of Japan. The Animal Subjects Committee at the National Cerebral and Cardiovascular Center reviewed and approved the experimental protocols (21009, 22033).

### Preparation

GK rats were purchased from Japan SLC and used in vehicle and empagliflozin groups (n = 7 each). The ages at the experiment were not significantly different between the two groups (vehicle: 16.2 ± 4.2 vs. empagliflozin: 17.8 ± 1.9 weeks, P = 0.456 by unpaired t-test). Rats were anesthetized with an intraperitoneal injection (2 mL kg^−1^) of a mixture of urethane (250 mg mL^−1^) and α-chloralose (40 mg mL^−1^). After induction of anesthesia, the tail artery was punctured with a 27G needle, and the blood glucose level was measured using a commercial glucose meter (FreeStyle Freedom Lite, Nipro, Japan). The anesthetic mixture was diluted 18-fold with physiological saline and infused continuously via the right femoral vein (2 mL·kg^−1^·h^−1^). Ringer’s lactate solution was infused continuously (4 mL·kg^−1^·h^−1^) for fluid maintenance. The rat was ventilated mechanically with oxygen-enriched air. AP was measured from the right femoral artery. Heart rate (HR) was detected from the AP waveform through a cardiotachometer (AT-601G, Nihon Kohden, Japan). The body temperature of the rat was maintained between 37 and 38 °C using a heating pad and a lamp.

For recording of sympathetic nerve activity (SNA), a postganglionic branch of the splanchnic sympathetic nerve was exposed through a left flank incision. A pair of stainless-steel wire electrodes (AS633, Cooner Wire, CA, USA) was attached to the nerve and fixed with silicone glue (Kwik-Sil, World Precision Instruments, FL, USA). The electrical signal was band-pass filtered between 150 and 1000 Hz, full-wave rectified, and low-pass filtered at a cut-off frequency of 30 Hz. The noise level of SNA was determined after an intravenous injection of a ganglionic blocker, hexamethonium bromide (60 mg kg^−1^), at the end of the experiment.

The bilateral carotid sinus baroreceptor regions were isolated from the systemic circulation [13, 14], and carotid sinus pressure (CSP) was externally regulated using a servo-controlled piston pump system (ET-126, Labworks, CA, USA). Reflexes other than the carotid sinus baroreflex were minimized by sectioning the aortic depressor and vagal nerves in the neck region.

For urine sampling, each ureter was cannulated with a polyethylene tube (KN-392-SP 8, Natsume, Japan) through a horizontal abdominal incision. Urine volume (UV) was assessed either from the hydrostatic pressure of urine accumulated in a vertically placed 1-mL syringe [15] or from the weight of the urine measured using an electronic balance (HR-150AZ, A&D Company, Japan) [16].

### Protocol

CSP was decreased to 60 mmHg for a period of 5 min and then increased stepwise up to 180 mmHg in increments of 20 mmHg every minute. The 11-min stepwise CSP input sequence was repeated and denoted as S1 through S8. Empagliflozin (MedChemExpress, NJ, USA) was dissolved in dimethyl sulfoxide (DMSO) at 10 mg/100 μL and diluted with polyethylene glycol and physiological saline to a final concentration of 10 mg mL^−1^ (10% v/v DMSO, 45% v/v polyethylene glycol 200, and 45% v/v physiological saline). One minute after completion of S2, the empagliflozin solution was administered intravenously at 10 mg kg^−1^ (1 mL kg^−1^, bolus). In the vehicle group, the DMSO solution not containing empagliflozin was administered.

### Data analysis

The CSP, SNA, AP, HR, and UV data were stored on a laboratory computer system at 1000 Hz via a 16-bit analog-to-digital converter. In each of the S1–S8 sequences, SNA, AP, and HR values were averaged during the last 10 s of each step. Urine from two consecutive sequences were combined for measurements of urine glucose and creatinine concentrations. Hence, hemodynamic data were also averaged for S1 and S2, S3 and S4, S5 and S6, and S7 and S8, which are referred to as baseline (BL), T1, T2, and T3, respectively.

The SNA was normalized in each animal between 0 and 100% because the absolute amplitude of SNA varied significantly across animals depending on recording conditions. The value after the ganglionic blockade was defined as 0%, and the value corresponding to the CSP of 60 mmHg during BL was defined as 100%.

The static characteristics of the baroreflex total arc, HR control, and neural arc were quantified using a four-parameter logistic function (Eq. 1) [17, 18],

1 $$y=\frac{{P}_{1}}{1+exp\left[{P}_{2}\left(CSP-{P}_{3}\right)\right]}+{P}_{4}$$

where *y* is AP, HR, or SNA; *P*_1_ is the response range; *P*_2_ is the slope coefficient; *P*_3_ is the midpoint pressure on the CSP axis; and *P*_4_ is the lower asymptote of the sigmoid curve. The maximum gain was calculated as *G*_max_ = *P*_1_ × *P*_2_/4.

The static characteristics of the baroreflex peripheral arc were quantified using linear regression (Eq. 2) [18],

2 $$AP={b}_{0}+{b}_{1}\times SNA$$

where *b*_0_ and *b*_1_ denote the intercept and slope, respectively.

The baroreflex equilibrium diagram combines the neural and peripheral arcs on a pressure versus SNA plane [19, 20]. The operating point was determined from the intersection of fitted neural and peripheral arcs on the equilibrium diagram.

The urine flow (UF, in μL min^−1^) during each step was calculated from an increment of UV summed from bilateral ureters. The normalized UF (nUF, in μL·min^−1^·kg^−1^) was defined as the UF divided by the body weight of the rat.

The relationship between AP and nUF during stepwise changes in CSP approximated a straight line in our previous studies [15, 21, 22]. For compatibility with those studies, the AP–nUF relationship was assessed as the relationship for a single kidney by halving the measured nUF. The intercept (nUF-intercept) and the slope (nUF-slope) of the AP–nUF relationship were estimated using linear regression.

Creatinine clearance (C_cr_) for a single kidney was calculated using nUF, the urine creatinine concentration obtained in each of BL, T1, T2, and T3 periods, and the plasma creatinine concentration measured at the end of the experiment, on the assumption that the plasma creatinine concentration had not changed significantly during the experiment. The nUF value corresponding to the AP at the operating point was estimated on the AP–nUF relationship and used to estimate C_cr_.

### Blood and urine samples

An arterial blood sample was obtained once at the end of the experiment. After centrifugation, the plasma sample was frozen at − 80 °C. Urine samples obtained during BL, T1, T2, and T3 periods were also frozen at − 80 °C. Later, the glucose and creatinine concentrations were measured by outsourcing (SRL Inc., Japan).

### Statistical analysis

All data are expressed as mean ± SE values. The plasma glucose and creatinine concentrations were compared between the vehicle and empagliflozin groups using unpaired t-tests [23]. The time-dependent changes in averaged nUF, urine glucose and creatinine concentrations, urine glucose excretion, C_cr_, nUF-intercept, and nUF-slope were analyzed using repeated-measures one-way analysis of variance (ANOVA) with the Greenhouse–Geisser correction, followed by a Dunnett's test (Prism 8, GraphPad Software, CA, USA). For the parameters of the baroreflex function, the comparison was made between BL and T3 periods using paired t-tests because the volume loading effect from a drug administration nearly diminished during T3 in the vehicle group. In all the statistical analyses, the differences were considered significant at P < 0.05.

## Results

The blood glucose levels measured by a tail artery puncture at the beginning of the experiment were not significantly different between the vehicle and empagliflozin groups (204.7 ± 4.6 vs. 213.6 ± 10.5 mg dL^−1^, P = 0.513).

**Fig. 1:**
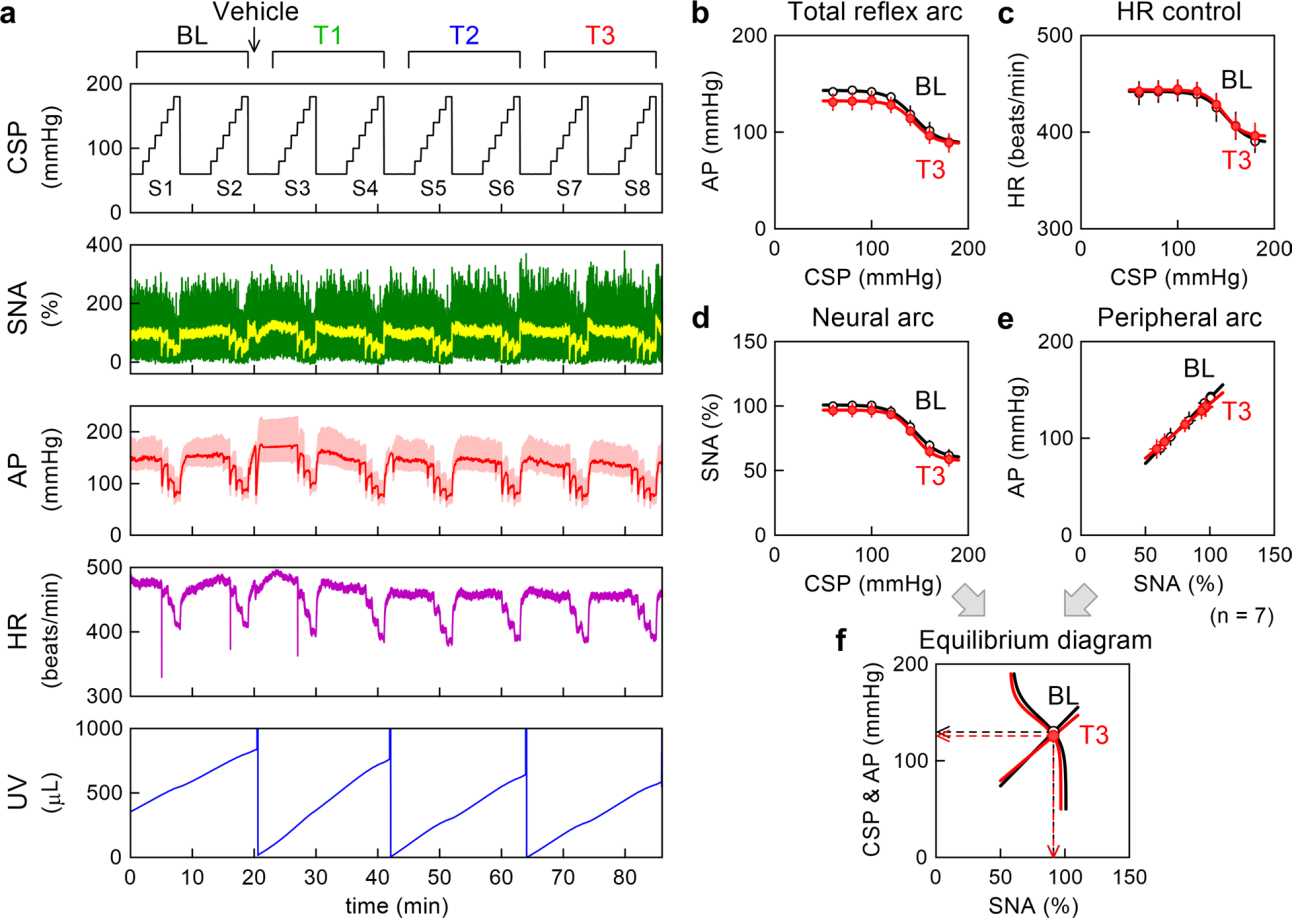
**a** An example time series obtained from a Goto–Kakizaki rat in the vehicle group. Carotid sinus pressure (CSP), sympathetic nerve activity (SNA), arterial pressure (AP), heart rate (HR), and urine volume (UV) during stepwise input sequences (S1–S8) are shown. In the CSP, HR, and UV plots, the data illustrate 10-Hz resampled signals. In the SNA plot, 10-Hz resampled (green) and 2-s moving averaged (yellow) signals are shown. In the AP plot, 100-Hz resampled (pale red) and 2-s moving averaged (red) signals are shown. In the UV plot, sharp reductions with vertical artifacts indicate manual urine removals. The data were divided into baseline (BL), T1, T2, and T3 periods of two input sequences each. The vehicle solution was administered one minute after the completion of S2. **b**–**e** Pooled data showing the total reflex arc, HR control, neural arc, and peripheral arc. The black lines with open circles represent the data obtained during BL. The red lines with filled circles represent the data obtained during T3. The data points represent mean ± SE values (n = 7 rats). **f** Baroreflex equilibrium diagrams derived from fitted neural and peripheral arcs. The black open circle and the red filled circle indicate the operating points during BL and T3, respectively. The leftward arrowheads indicate the AP values at the operating points. The downward arrowheads indicate the SNA values at the operating points

Figure 1 illustrates an example time series (Fig. 1a) as well as pooled data of the baroreflex open-loop static characteristics (Fig. 1b–f) obtained in the vehicle group. In the time series, a stepwise increase in CSP suppressed SNA and decreased AP and HR. The administration of the vehicle solution increased AP during T1 (mainly S3) compared with BL, but the effect diminished during T3. Although HR showed a decreasing trend in this rat, the observation was not consistent across the rats. UV increased with time. Abrupt vertical changes in the UV plot were artifacts caused by manual urine removals. The vehicle solution slightly steepened the increasing slope of UV during T1 compared with BL, but the steepening effect was not obvious during T3.

In the pooled data, the mean line of the total reflex arc showed a slight downward displacement during low CSP levels with a marginal narrowing of the response range when compared between BL and T3 (Fig. 1b, Table 1). None of the HR control (Fig. 1c), neural arc (Fig. 1d), and peripheral arc (Fig. 1e) significantly changed after the administration of the vehicle solution. In the baroreflex equilibrium diagram (Fig. 1f), neither the AP values at the operating point (the leftward arrowheads) nor the SNA values at the operating point (the downward arrowheads) differed significantly between BL and T3 (Table 1).

**Table 1:** Parameters relating to baroreflex open-loop static characteristics during baseline and T3 periods obtained in the vehicle group

	Baseline	Vehicle	P value
Total reflex arc			
*P*_1_, mmHg	54.6 ± 6.2	45.6 ± 9.1	0.061
*P*_2_, mmHg^−1^	0.120 ± 0.018	0.106 ± 0.013	0.471
*P*_3_, mmHg	145.5 ± 5.2	143.5 ± 1.8	0.745
*P*_4_, mmHg	88.4 ± 6.2	87.1 ± 9.5	0.776
*G*_max_, mmHg/mmHg	1.62 ± 0.28	1.05 ± 0.13	0.065
HR control			
*P*_1_, beats/min	53.5 ± 6.4	50.2 ± 9.7	0.473
*P*_2_, mmHg^−1^	0.115 ± 0.013	0.131 ± 0.022	0.418
*P*_3_, mmHg	151.0 ± 3.9	148.3 ± 2.1	0.549
*P*_4_, beats/min	389.1 ± 10.8	394.1 ± 13.0	0.672
*G*_max_, beats·min^−1^·mmHg^−1^	1.49 ± 0.17	1.34 ± 0.14	0.342
Neural arc			
*P*_1_, %	41.3 ± 4.6	40.4 ± 7.3	0.899
*P*_2_, mmHg^−1^	0.137 ± 0.028	0.121 ± 0.022	0.665
*P*_3_, mmHg	143.6 ± 6.1	143.1 ± 3.4	0.947
*P*_4_, %	59.7 ± 3.9	57.1 ± 5.2	0.553
*G*_max_, %/mmHg	1.34 ± 0.23	1.03 ± 0.08	0.113
Peripheral arc			
*b*_0_, mmHg	16.6 ± 9.6	28.0 ± 11.1	0.303
*b*_1_, mmHg/%	1.245 ± 0.101	1.109 ± 0.084	0.234
Operating point			
SNA, %	92.1 ± 2.1	88.3 ± 3.2	0.448
AP, mmHg	130.2 ± 4.0	126.8 ± 2.5	0.385

*P*_1_: response range; *P*_2_: slope coefficient; *P*_3_: midpoint input pressure; *P*_4_: lower asymptote; *G*_max_: maximum gain; *b*_0_: intercept; *b*_1_: slope; HR: heart rate; SNA: sympathetic nerve activity; AP: arterial pressure. Data are mean ± SE values (n = 7 rats). P values are derived from paired t-tests

**Fig. 2:**
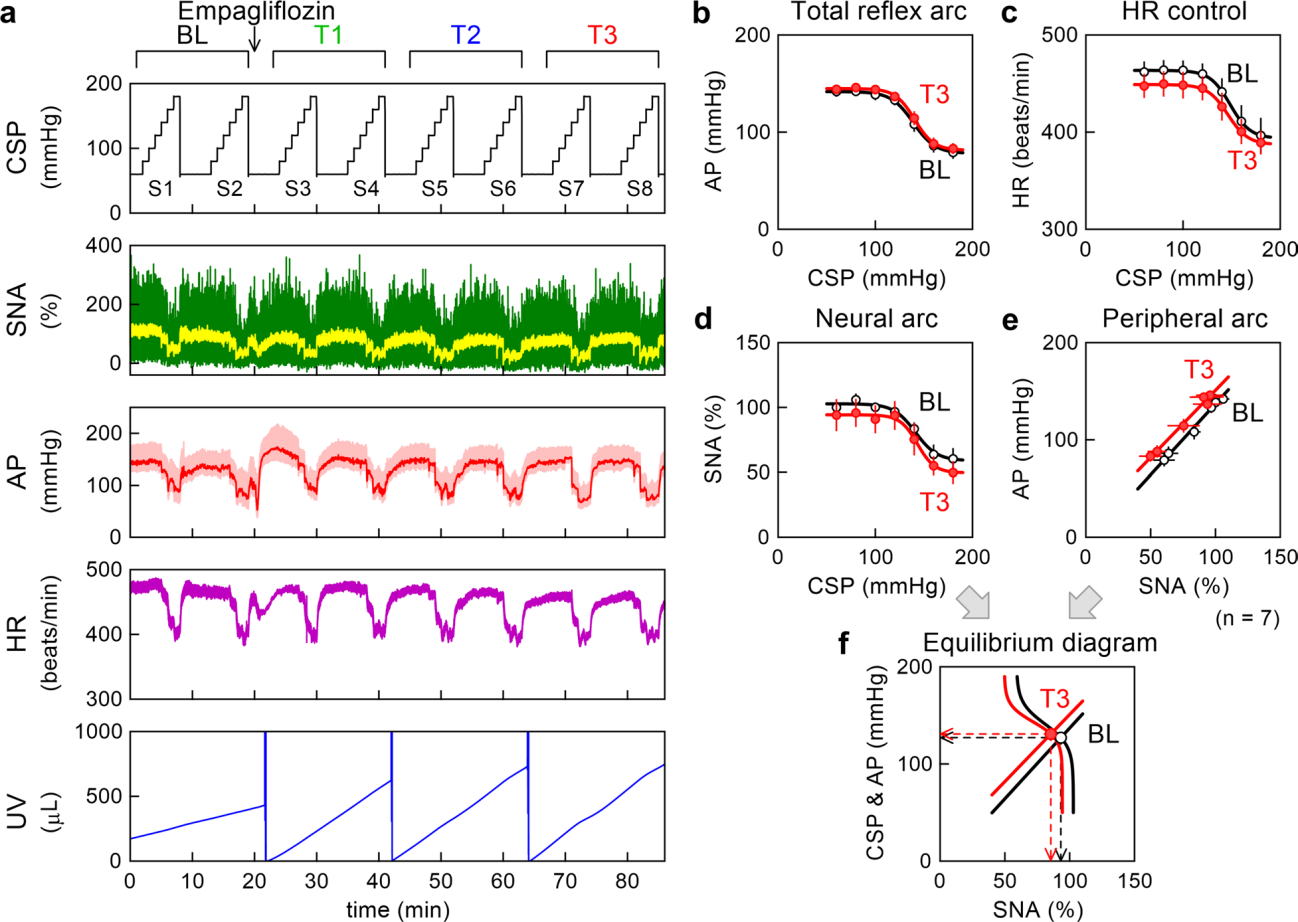
**a** An example time series obtained from a Goto–Kakizaki rat in the empagliflozin group. Carotid sinus pressure (CSP), sympathetic nerve activity (SNA), arterial pressure (AP), heart rate (HR), and urine volume (UV) during stepwise input sequences (S1–S8) are shown. In the CSP, HR, and UV plots, the data illustrate 10-Hz resampled signals. In the SNA plot, 10-Hz resampled (green) and 2-s moving averaged (yellow) signals are shown. In the AP plot, 100-Hz resampled (pale red) and 2-s moving averaged (red) signals are shown. In the UV plot, sharp reductions with vertical artifacts indicate manual urine removals. The data were divided into baseline (BL), T1, T2, and T3 periods of two input sequences each. Empagliflozin (10 mg kg^−1^) was administered one minute after the completion of S2. **b**–**e** Pooled data showing the total reflex arc, HR control, neural arc, and peripheral arc. The black lines with open circles represent the data obtained during BL. The red lines with filled circles represent the data obtained during T3. The data points represent mean ± SE values (n = 7 rats). **f** Baroreflex equilibrium diagrams derived from fitted neural and peripheral arcs. The black open circle and the red filled circle indicate the operating points during BL and T3, respectively. The leftward arrowheads indicate the AP values at the operating points. The downward arrowheads indicate the SNA values at the operating points

Figure 2 illustrates an example time series (Fig. 2a) and pooled data of the baroreflex open-loop static characteristics (Fig. 2b–f) obtained in the empagliflozin group. A stepwise increase in CSP suppressed SNA and decreased AP and HR. Empagliflozin increased AP during T1 (mainly S3), but the effect subsided during T3. HR showed a slight decreasing trend. Empagliflozin steepened the increasing slope of UV during T1, T2, and T3 compared with BL.

In the pooled data, empagliflozin did not affect the total reflex arc (Fig. 2b, Table 2). Although mean lines of the HR control (Fig. 2c) and neural arc (Fig. 2d) showed a slight downward displacement, the fitted parameters were not significantly different between BL and T3. Although the mean line of the peripheral arc (Fig. 2e) showed a slight upward displacement, neither the intercept nor the slope was significantly different between BL and T3. In the baroreflex equilibrium diagram (Fig. 2f), empagliflozin did not significantly affect the AP or SNA value at the operating point (Table 2).

**Table 2:** Parameters relating to baroreflex open-loop static characteristics during baseline and T3 periods obtained in the empagliflozin group

	Baseline	Empagliflozin	P value
Total reflex arc			
*P*_1_, mmHg	64.2 ± 7.5	64.0 ± 4.9	0.982
*P*_2_, mmHg^−1^	0.106 ± 0.010	0.111 ± 0.016	0.832
*P*_3_, mmHg	139.9 ± 3.5	140.5 ± 2.8	0.877
*P*_4_, mmHg	78.1 ± 5.9	81.2 ± 5.0	0.282
*G*_max_, mmHg/mmHg	1.66 ± 0.24	1.78 ± 0.30	0.779
HR control			
*P*_1_, beats/min	68.8 ± 9.6	60.7 ± 7.1	0.271
*P*_2_, mmHg^−1^	0.106 ± 0.008	0.116 ± 0.008	0.324
*P*_3_, mmHg	148.1 ± 2.8	144.9 ± 3.4	0.367
*P*_4_, beats/min	395.0 ± 17.4	388.5 ± 11.2	0.504
*G*_max_, beats·min^−1^·mmHg^−1^	1.82 ± 0.25	1.72 ± 0.15	0.642
Neural arc			
*P*_1_, %	44.7 ± 6.3	44.7 ± 9.6	0.998
*P*_2_, mmHg^−1^	0.145 ± 0.027	0.167 ± 0.031	0.667
*P*_3_, mmHg	138.1 ± 6.7	139.2 ± 3.9	0.810
*P*_4_, %	57.3 ± 6.4	49.1 ± 6.7	0.090
*G*_max_, %/mmHg	1.51 ± 0.19	1.85 ± 0.43	0.351
Peripheral arc			
*b*_0_, mmHg	5.0 ± 9.3	8.3 ± 16.7	0.820
*b*_1_, mmHg/%	1.320 ± 0.115	1.527 ± 0.223	0.344
Operating point			
SNA, %	92.0 ± 2.5	85.2 ± 9.2	0.457
AP, mmHg	125.7 ± 4.2	130.3 ± 2.4	0.224

*P*_1_: response range; *P*_2_: slope coefficient; *P*_3_: midpoint input pressure; *P*_4_: lower asymptote; *G*_max_: maximum gain; *b*_0_: intercept; *b*_1_: slope; HR: heart rate; SNA: sympathetic nerve activity; AP: arterial pressure. Data are mean ± SE values (n = 7 rats). P values are derived from paired t-tests

**Fig. 3:**
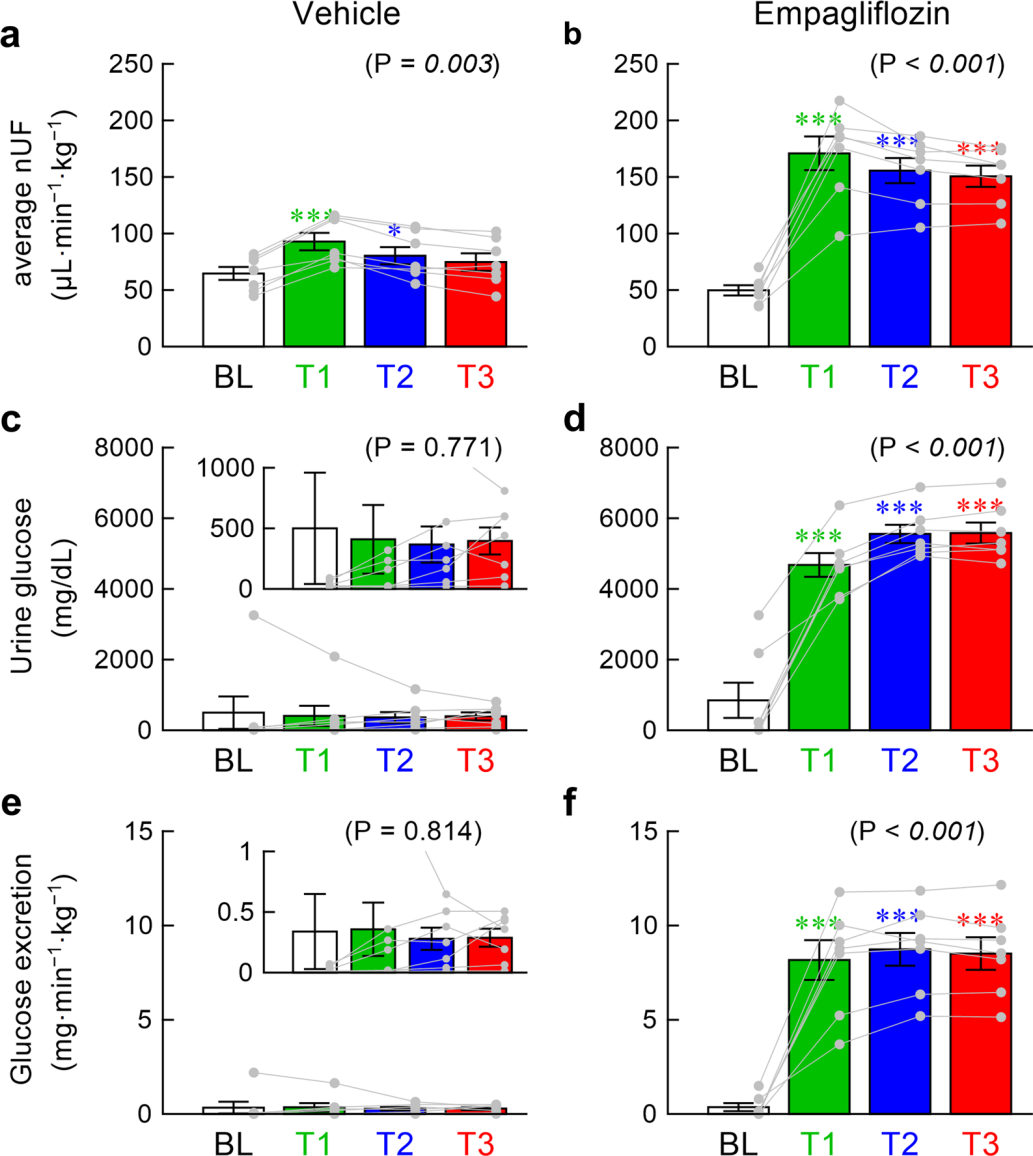
The top panels illustrate the averaged normalized urine flow (nUF) during baseline (BL), T1, T2, and T3 periods obtained in the vehicle (**a**) and empagliflozin (**b**) groups. The middle panels illustrate the urine glucose concentration during BL, T1, T2, and T3 periods obtained in the vehicle (**c**) and empagliflozin (**d**) groups. The bottom panels illustrate the urine glucose excretion during BL, T1, T2, and T3 periods obtained in the vehicle (**e**) and empagliflozin (**f**) groups. Data are presented as mean ± SE values with data points of the respective rats (n = 7 rats each). In panels **c**, **e**, the insets indicate the enlarged ordinates. In each panel, the P value of the repeated-measures one-way analysis of variance (ANOVA) is shown in the parenthesis. The value is italicized when P < 0.05. Symbols * and *** indicate P < 0.05 and P < 0.001 from BL by the post hoc Dunnett’s test

The vehicle solution significantly increased the averaged nUF during T1 and T2 (Fig. 3a), but the absolute increase was much smaller than that observed after the administration of empagliflozin (Fig. 3b). The nUF during T3 was not significantly different from that during BL in the vehicle group, whereas it remained increased in the empagliflozin group. The vehicle solution did not affect the urine glucose concentration (Fig. 3c). In the enlarged ordinate (Fig. 3c, inset), six rats showed an increasing trend in the urine glucose concentration. When the six rats were selected, the increasing trend was statistically significant as an overall effect (P = 0.028), but the increase in each point relative to BL was not statistically significant by post hoc analysis. Empagliflozin markedly increased the urine glucose concentration (Fig. 3d). The urine glucose excretion did not change significantly in the vehicle group (Fig. 3e) but markedly increased in the empagliflozin group (Fig. 3f). The plasma glucose concentration measured at the end of the experiment was significantly lower in the empagliflozin than in the vehicle group (286.4 ± 23.6 vs. 486.0 ± 37.1 mg dL^−1^, P < 0.001).

**Fig. 4:**
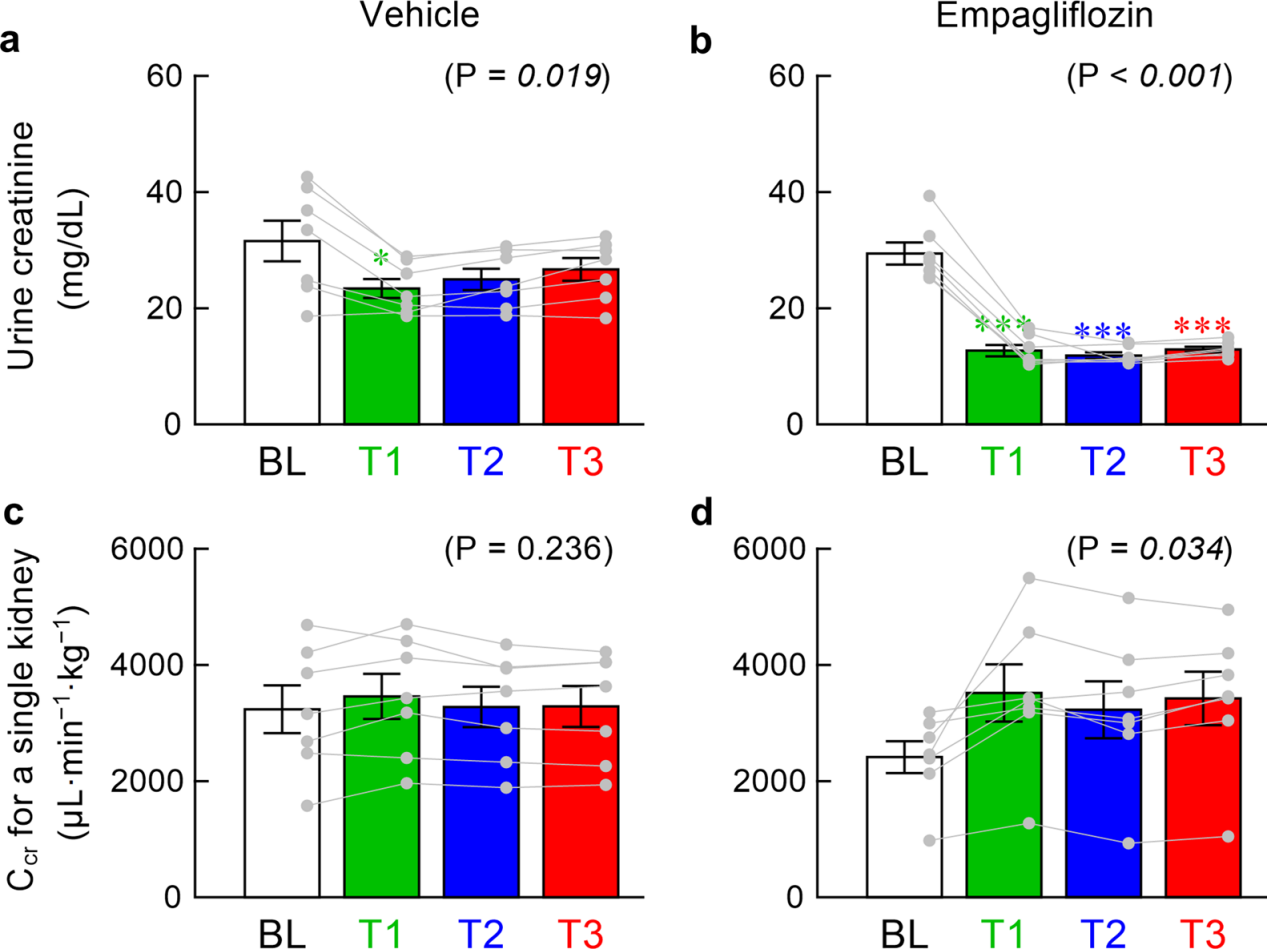
The upper panels illustrate the urine creatinine concentration during baseline (BL), T1, T2, and T3 periods obtained in the vehicle (**a**) and empagliflozin (**b**) groups. The lower panels illustrate the creatinine clearance (C_cr_) for a single kidney during BL, T1, T2, and T3 periods obtained in the vehicle (**c**) and empagliflozin (**d**) groups. Data are presented as mean ± SE values with data points of the respective rats (n = 7 rats each). In each panel, the P value of the repeated-measures one-way analysis of variance (ANOVA) is shown in the parenthesis. The value is italicized when P < 0.05. Symbols * and *** indicate P < 0.05 and P < 0.001 from BL by the post hoc Dunnett’s test

The plasma creatinine concentrations measured at the end of the experiment were not significantly different between the vehicle and empagliflozin groups (0.323 ± 0.024 vs. 0.333 ± 0.048 mg mL^−1^, P = 0.854). Although the vehicle solution reduced the urine creatinine concentration during T1 compared with BL, the effect was not significant during T2 or T3 (Fig. 4a). Empagliflozin significantly decreased the urine creatinine concentration during T1, T2, and T3 (Fig. 4b). C_cr_ in the vehicle group showed a large inter-individual difference and did not show significant time-dependent changes (Fig. 4c). Although empagliflozin increased C_cr_ as an overall effect, the increase in each point relative to BL was not statistically significant by post hoc analysis (Fig. 4d).

**Fig. 5:**
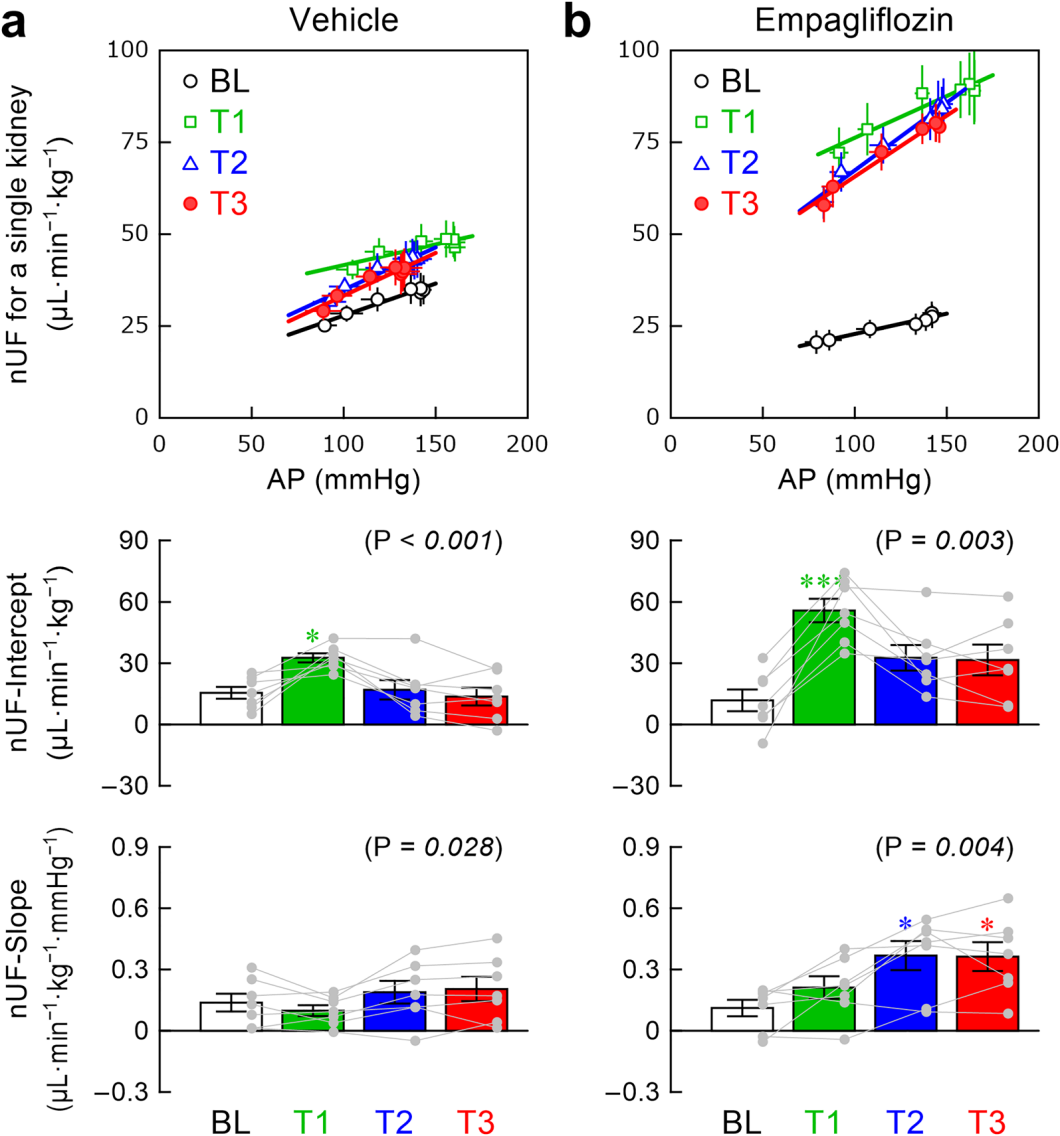
The relationships of normalized urine flow (nUF) versus arterial pressure (AP) during baseline (BL), T1, T2, and T3 periods obtained in the vehicle (**a**) and empagliflozin (**b**) groups. The top panels show mean ± SE values with linear regression lines. In the middle panels, nUF-intercept indicates the intercept of the AP–nUF relationship. In the bottom panels, nUF-slope indicates the slope of the AP–nUF relationship. For nUF-intercept and nUF-slope, the data are presented as mean ± SE values with data points of the respective rats (n = 7 rats each). In each panel, the P value of the repeated-measures one-way analysis of variance (ANOVA) is shown in the parenthesis. The value is italicized when P < 0.05. Symbols * and *** indicate P < 0.05 and P < 0.001 from BL by the post hoc Dunnett’s test

The relationship between AP and nUF during the stepwise changes in CSP approximated a straight line in both the vehicle (Fig. 5a) and empagliflozin (Fig. 5b) groups. In the vehicle group, the nUF-intercept increased during T1 but returned toward the BL level during T2 and T3. The change in the nUF-slope was significant only as an overall effect. In the empagliflozin group, the nUF-intercept significantly increased during T1, but the increase was not statistically significant during T2 and T3. The nUF-slope increased after empagliflozin, and the increasing effect was statistically significant during T2 and T3 compared with BL.

## Discussion

The acute effects of an intravenous administration of empagliflozin on baroreflex function and urine glucose excretion were examined in GK rats. Although empagliflozin increased urine glucose excretion with nearly tripled nUF (Fig. 3), it did not significantly modify the baroreflex neural or peripheral arc during T3 compared with BL (Fig. 2, right panels).

### Effects of empagliflozin on baroreflex function in GK rats

Although an AP reduction with diuretics is usually accompanied by reflex tachycardia, reflex tachycardia is not observed during treatment with SGLT2 inhibitors. The AP reduction after a 4-day treatment with empagliflozin was not associated with an increase in muscle SNA in diabetic patients, indicating a sympathoinhibitory effect of empagliflozin [7]. Renal metabolism is related to the development of hypertension [24]. The inhibition of glucose reabsorption may reduce the energy burden at proximal tubules in DM and attenuate sympathoexcitatory reflexes arising from the kidneys [2]. However, empagliflozin did not significantly alter the neural arc despite the marked increase in urine glucose excretion (Figs. 2d, 3f). Hence, changes in renal energy status did not acutely modify the baroreflex-mediated systemic sympathetic regulation, at least, in the present experimental settings.

The neural effect of empagliflozin contrasts with that of clonidine, a central antihypertensive, examined in our previous study using the baroreceptor isolation procedure [25]. In that study, an intravenous administration of clonidine hydrochloride at 5 μg kg^−1^ (a cumulative dose of 7 μg kg^−1^) immediately suppressed SNA (the response range was narrowed from 93.9 ± 2.4 to 24.0 ± 6.2%, P < 0.001). Considering that the molecular weight of empagliflozin (450.91) is only twice as large as clonidine hydrochloride (266.55), clonidine revealed a central sympathoinhibitory effect at a concentration far below that of empagliflozin. Although the strain difference existed [GK in the present study versus Wistar–Kyoto (WKY) rats in our previous study], the acute sympathoinhibitory effect of empagliflozin was minimum, if any, compared with clonidine.

A previous study demonstrated that a two-week treatment with dapagliflozin decreased the norepinephrine content in the kidneys in high-fat fed mice [26]. Gueguen et al. [27] demonstrated that one-week treatment with empagliflozin attenuated the DM-induced augmentation of the maximum renal SNA in rabbits, but the effect was not observed with a single dose. Gueguen et al. [27] indicate that the change in SNA induced by empagliflozin likely represents a realignment of renal function rather than a direct (instantaneous) effect of the drug on SNA.

Urine output nearly tripled after the administration of empagliflozin (Fig. 3b). The averaged nUF was approximately 9 mL·kg^−1^·h^−1^ during T3, which exceeded the fluid infusion rate of 6 mL·kg^−1^·h^−1^ (4 mL·h^−1^·kg^−1^ of Ringer’s lactate solution plus 2 mL·h^−1^·kg^−1^ of anesthetics). Although the negative fluid balance should have reduced intravascular volume, the peripheral arc did not change significantly (Fig. 2e). Because the bolus administration of empagliflozin at 1 mL kg^−1^ partly offset the volume loss, the observation period might have been too short to detect an expected reduction in the peripheral arc.

A differential volume regulation between interstitial fluid volume and intravascular fluid volume by SGLT2 inhibitors is postulated based on a mathematical model analysis [28]. SGLT2 inhibitors can retain intravascular volume compared with loop diuretics [5]. One possible explanation for the maintenance of intravascular volume is that SGLT2 inhibitors reduce the insulin:glucagon ratio that increases hepatic glucose production [29, 30]. As the water moves with glucose [31], the increased hepatic glucose production can move the water from the interstitial space or cell to the vessels. Although further studies are required to elucidate the magnitude and the time course of this fluid movement, such a mechanism might have also partly contributed to the maintenance of AP in the face of increased urine output.

### Effects of empagliflozin on urine glucose excretion and creatinine clearance

Empagliflozin increased the urine output and urine glucose excretion from T1, and the increasing effects continued until T3 (Fig. 3b, f). The dose of empagliflozin was sufficiently high to reduce the plasma glucose concentration at the end of the experiment in the empagliflozin group compared with the vehicle group. The study on pharmcodynamics of SGLT2 inhibitors in mice indicates that an oral administration of empagliflozin (up to 10 mg kg^−1^) decreased the plasma glucose concentration with a nadir at approximately 1 h [32]. Since an injection of glucose solution to the lateral ventricles of the brain increases SNA in rats [33], it is possible that the reduction of the plasma glucose concentration attenuates sympathetic activation. A reduction of the plasma insulin level after empagliflozin [32] may also be a possible factor to reduce SNA because insulin acts as a sympathoexcitatory hormone [34]. Nevertheless, no significant changes in the neural arc (Fig. 2d) indicate that the magnitude of the change in the plasma glucose or insulin level was too small to yield a significant effect on SNA.

Empagliflozin significantly increased C_cr_ as an overall effect (Fig. 4d). However, a somewhat lower distribution of C_cr_ during BL in the empagliflozin than in the vehicle group (Fig. 4c) makes the effect of empagliflozin on C_cr_ inconclusive. Regarding another SGLT2 inhibitor ipragliflozin, C_cr_ decreased after the intravenous drug administration in spontaneously diabetic Torii fatty rats [35]. C_cr_ during BL in the empagliflozin group (2414 ± 276 μL·min^−1^·kg^−1^, n = 7) is not significantly different from C_cr_ of the intact kidney in WKY rats (2662 ± 352 μL·min^−1^·kg^−1^, n = 9, P = 0.605 by unpaired t-test) [15] estimated under similar experimental settings. In a developmental study, C_cr_ of GK rats is not different from C_cr_ of Wistar rats at 2 months of age, and significantly decreases at 8 and 14 months of age [36]. The GK rats used in the present study (4–4.5 months of age) might have still maintained a near normal glomerular filtration rate.

### Relationship between AP and nUF during baroreflex mediated changes in AP

Under baroreflex open-loop conditions, baroreflex-mediated changes in SNA yield a positive relationship between SNA and AP in the peripheral arc (Figs. 1e, 2e). An increase in AP promotes urine excretion through pressure diuresis, whereas sympathetic activation exerts antidiuretic effect through renin release, renal vasoconstriction, and sodium reabsorption [37]. If the neurally-mediated antidiuretic effect is more powerful than the pressure diuresis effect, urine excretion should decrease as SNA increases. However, urine excretion increased as SNA increased in our previous studies using WKY rats, indicating that the pressure diuresis effect outweighs the neurally-mediated antidiuretic effect [15, 16, 22].

In the present study, nUF-slope was near zero in two rats in the vehicle group (Fig. 5a) and negative in two rats in the empagliflozin group (Fig. 5b), indicating an impairment of the pressure diuresis effect and/or an enhancement of the neurally-mediated antidiuretic effect in GK rats. The nUF-slope during BL in the empagliflozin group (0.111 ± 0.040 μL·min^−1^·kg^−1^·mmHg^−1^, n = 7) is significantly lower than nUF-slope of the intact kidney in WKY rats (0.420 ± 0.081 μL·min^−1^·kg^−1^·mmHg^−1^, n = 10, P = 0.005 by unpaired t-test) [15]. Hence, the urine output function seems to be significantly depressed in GK rats compared with WKY rats even at the age when C_cr_ is relatively maintained (see the previous section). A histological analysis on GK rat kidneys indicates that diffuse thickening of glomerular basement membrane occurs from 12 weeks of age and becomes more prominent at 16 to 24 weeks of age [38]. Such a histological change may account for the depressed nUF-slope in GK rats. Empagliflozin increased nUF-slope toward the value of nUF-slope in WKY rats (Fig. 5b). In addition to an increase in averaged nUF (Fig. 3b), the increased nUF-slope after empagliflozin may promote the fluid volume control because nUF-slope is a system gain of urine output control in response to changes in AP.

### Limitations

As we only examined the acute effects of empagliflozin under anesthetized conditions, the results may not be directly applicable to understand any chronic effect of empagliflozin. One difficulty in examining the chronic effect of a test drug on the sympathetic regulation is that the level of SNA is difficult to compare between different time points or between different groups because the absolute amplitude of SNA can be affected by recording conditions. When SNA is expressed in normalized units, a potential difference in SNA could be obscured. One way to circumvent the problem is to calibrate SNA against plasma noradrenaline concentrations [39]. However, if chronic improvements of disease conditions attenuate sympathetic activation and reduce the plasma noradrenaline concentrations, we will remain unsure about whether the change in SNA is attributable to a direct sympathoinhibitory effect of the test drug.

## Conclusions

The sympathetic suppression via the SGLT2 inhibition was not detected within the observation period despite the significant increase in urine glucose excretion and the reduction of plasma glucose concentration in the present experimental settings. It would take some time for the manifestation of possible sympathetic suppression after the SGLT2 inhibition, and the sympathoinhibitory effect may be an indirect effect associated with chronic improvements of renal energy status and general disease conditions.

## Data Availability

The datasets used and/or analyzed during the current study are available from the corresponding author on reasonable request.

## Abbreviations

AP: Arterial pressure
BL: Baseline
C_cr_: Creatinine clearance
CSP: Carotid sinus pressure
DM: Diabetic mellitus
DSMO: Dimethyl sulfoxide
GK: Goto–Kakizaki
HR: Heart rate
nUF: Normalized urine flow
SGLT2: Sodium–glucose cotransporter 2
SNA: Sympathetic nerve activity
UF: Urine flow
UV: Urine volume
WKY: Wistar–Kyoto
